# Assessment of an Online Tool to Simulate the Effect of Pooled Testing for SARS-CoV-2 Detection in Asymptomatic and Symptomatic Populations

**DOI:** 10.1001/jamanetworkopen.2020.31517

**Published:** 2020-12-10

**Authors:** Christopher R. Polage, Mark J. Lee, Christopher Hubbard, Catherine Rehder, Diana Cardona, Thomas Denny, Michael B. Datto

**Affiliations:** 1 Duke Health System Clinical Laboratories, Durham, North Carolina; 2 Department of Pathology, Duke University School of Medicine, Durham, North Carolina; 3 Department of Medicine, Duke Human Vaccine Institute, Duke University School of Medicine, Durham, North Carolina

## Abstract

This diagnostic study describes an online tool created with actual severe acute respiratory syndrome coronavirus 2 (SARS-CoV-2) virus copy number data to help policy makers understand how pooled testing compares with single-sample testing in different populations.

## Introduction

Policy makers are promoting pooled testing as a strategy to increase the number of people tested for the severe acute respiratory syndrome coronavirus 2 (SARS-CoV-2) during the coronavirus disease 2019 (COVID-19) pandemic, especially for population screening.^1,2^ However, combining samples before testing brings trade-offs, such as decreasing the sensitivity and increasing the complexity of testing, that should be considered.^3,4,5,6^ We created an online tool using actual SARS-CoV-2 virus copy number (VCN) data—the COVID19 Pool Tool—to help policy makers understand how pooled testing compares with single-sample testing in different populations.

## Methods

This diagnostic study was approved with a waiver of consent and Health Insurance Portability and Accountability Act authorization by the Duke Health institutional review board as part of retrospective SARS-CoV-2 test method comparison and validation studies. This study follows the 2015 Standards for Reporting of Diagnostic Accuracy (STARD) reporting guideline.

We used droplet digital polymerase chain reaction (PCR) to count SARS-CoV-2 copy numbers in patient samples and create quantitative curves for 3 US Food and Drug Administration Emergency Use Authorized SARS-CoV-2 reverse transcriptase–PCR qualitative tests. We used these curves to convert positive nasopharyngeal PCR cycle threshold (Ct) values to droplet digital PCR–harmonized VCNs for these tests. We created an online tool that allows users to define the sample size, pool size, positivity rate, and test characteristics and to compare pooled testing with single-sample testing using actual VCN data (the COVID19 Pool Tool; see the Additional Information at the end of the article for the URL). The tool uses VCNs from preprocedural screening samples (ie, no clinical suspicion for COVID-19) and outpatient diagnostic samples (ie, clinical suspicion for COVID-19) to simulate asymptomatic and symptomatic SARS-CoV-2–positive patients. The tool generates random virtual pools with positive samples pulled from VCN data mirroring the expected positivity rate and calculates pool VCNs with the expected dilution. The tool uses VCNs and user-defined test characteristics to predict which positive pools and samples are detected or missed, the sensitivity of pooled and single sample testing, and the number of tests performed. Additional details are shown in the eAppendix in the Supplement.

We used this tool to compare pooled testing with single-sample testing via a common reverse transcriptase–PCR assay (RealTime SARS-CoV-2 Assay; Abbott), with a 95% limit of detection of 100 VCNs/mL and an absolute limit of detection of 2 VCNs/mL, for detection of asymptomatic and symptomatic SARS-CoV-2–positive patients at various positivity rates. A screenshot of the COVID19 Pool Tool user interface is shown in eFigure 1 in the Supplement, and an example of COVID19 Pool Tool results is shown in eFigure 2 in the Supplement.

Calculation of VCNs from Ct values was performed using Excel software version 2016 (Microsoft). Figures were created in Prism software version 6.0c (GraphPad) and Excel. Data analysis was performed in August 2020 using data collected from March to July 2020.

## Results

**Figure 1.**
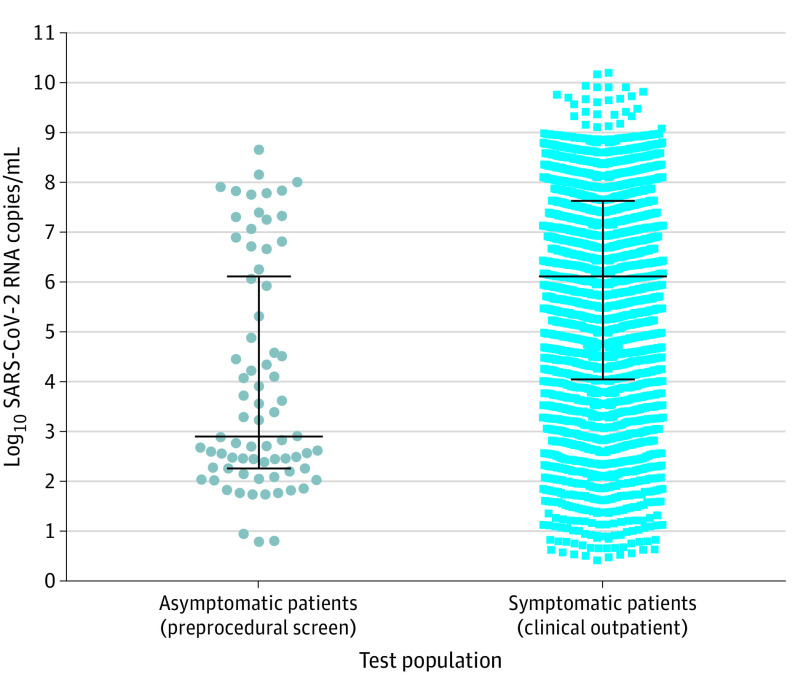
Virus Copy Number (VCN) Distribution of Asymptomatic and Symptomatic Severe Acute Respiratory Syndrome Coronavirus 2 (SARS-CoV-2)–Positive Patient Samples Used in the COVID19 Pool Tool Gray circles represent VCNs of 74 individual SARS-CoV-2–positive preprocedural screening samples (median [interquartile range] VCN, 2.90 [2.26-6.11] log_10_ RNA copies/mL). Blue squares represent VCNs of 2910 individual SARS-CoV-2-positive outpatient diagnostic samples (median [interquartile range] VCN, 6.11 [4.05-7.62] log_10_ RNA copies/mL).

**Figure 2.**
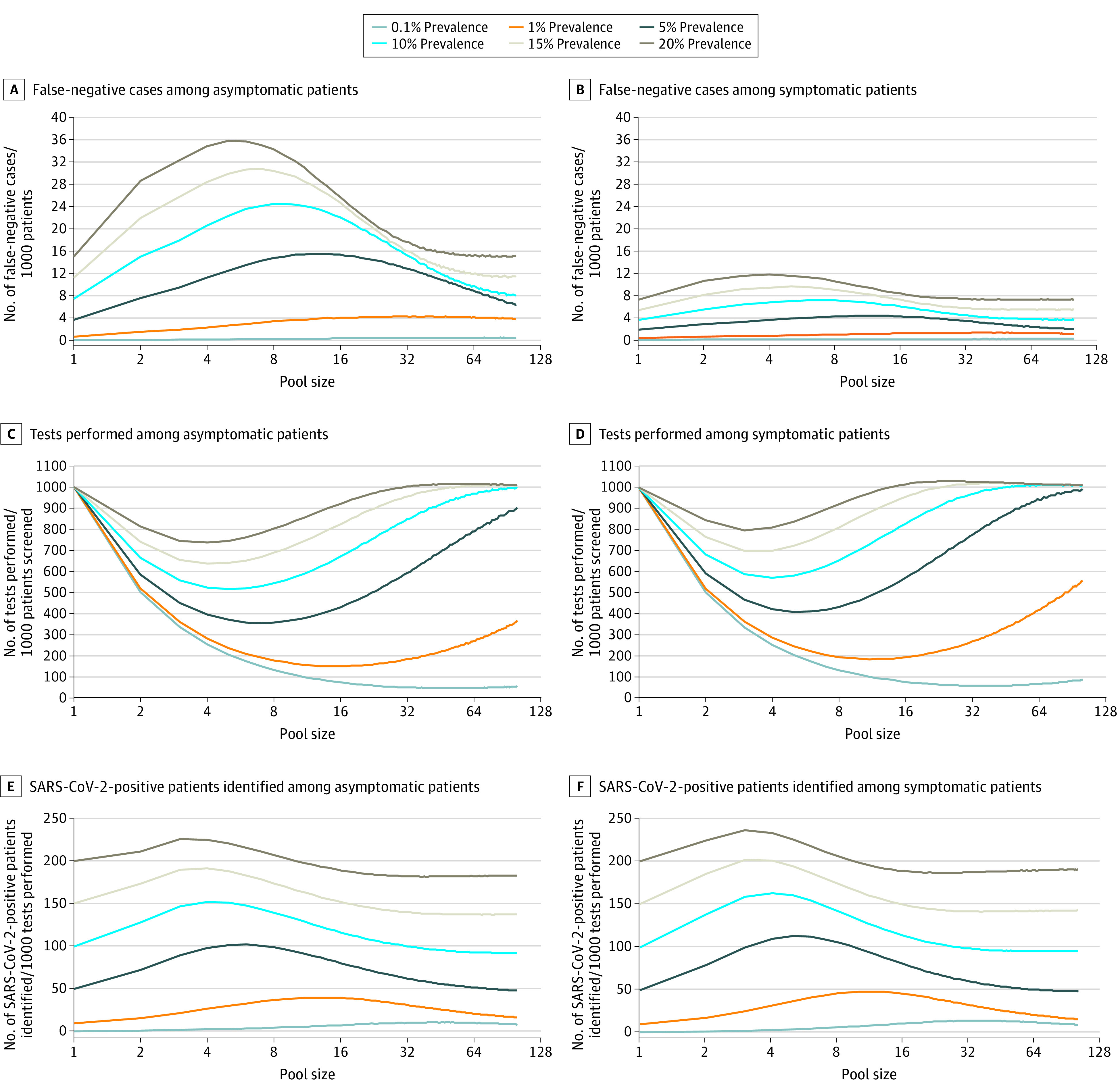
Performance of Pooled Testing for Detection of Asymptomatic and Symptomatic Severe Acute Respiratory Syndrome Coronavirus 2 (SARS-CoV-2)–Positive Patients With a High-Sensitivity Reverse Transcriptase–Polymerase Chain Reaction Assay The performance of single-sample testing is shown on the *y*-axis (pool size = 1) of each graph.

Figure 1 shows the 74 preprocedural screening sample VCNs (median [interquartile range] VCN, 2.90 [2.26-6.11] log_10_ RNA copies/mL) and 2910 outpatient diagnostic sample VCNs (median [interquartile range] VCN, 6.11 [4.05-7.62] log_10_ RNA copies/mL) used to simulate asymptomatic and symptomatic SARS-CoV-2–positive patients in the tool. Figure 2 compares pooled testing with single-sample testing across positivity rates. Pooled testing increased the number of false-negative cases per 1000 patients and decreased the sensitivity of SARS-CoV-2 detection, especially for low-VCN samples and asymptomatic patients (Figure 2A and B). For example, the mean (SD) sensitivity of 5-sample pooled testing for detection of asymptomatic patients with a 10% positivity rate in the population was 77.57% (4.40%) vs 92.83% (8.38%) for single-sample testing. Figure 2C and D show that pooled testing decreased the number of tests needed to screen 1000 patients, but this effect was reduced by repeat testing to identify cases from positive pools for contract tracing, especially at high prevalence. Figure 2E and F show that pooled testing identified more positive patients per 1000 tests performed by testing more patients, but repeat testing reduced this benefit.

## Discussion

Pooled testing can extend SARS-CoV-2 test supplies and increase the number of patients tested and cases detected, making it useful for population screening and resource-constrained settings. The complicated workflow, lower sensitivity for low-VCN patients, and need to repeat tests for positive pools are drawbacks. Sequential 2-stage pooling could reduce the burden of retesting from positive large pools (see Additional Information at the end of the article for the 2-stage COVID19 Pool Tool URL). The lack of clinical data for laboratory samples is a potential limitation of this study. This tool offers policy makers an easy to use tool to inform regional and national decision-making about the pros and cons of pooled testing.

## Notes

Supplement. **eAppendix.** Additional Methods for Study Population, Laboratory Testing, COVID19 Pool Tool, and Virtual Diagnostic Study Analysis **eReference** **eFigure 1.** Screenshot of COVID19 Pool Tool User Interface **eFigure 2.** Example COVID19 Pool Tool Results
