# Supplementary material for: Assessment of an Online Tool to Simulate the Effect of Pooled Testing for SARS-CoV-2 Detection in Asymptomatic and Symptomatic Populations
Source: JAMA Netw Open. 2020 Dec 10;3(12):e2031517. doi: 10.1001/jamanetworkopen.2020.31517 (PMC7729431; doi:10.1001/jamanetworkopen.2020.31517)
Supplement: Supplement. — eAppendix. Additional Methods for Study Population, Laboratory Testing, COVID19 Pool Tool, and Virtual Diagnostic Study Analysis eReference eFigure 1. Screenshot of COVID19 Pool Tool User Interface eFigure 2. Example COVID19 Pool Tool Results [file jamanetwopen-e2031517-s001.pdf]

Supplemental Online Content

Polage CR, Lee MJ, Hubbard C, et al. Assessment of an online tool to simulate the effect of pooled testing for SARS-CoV-2 detection in asymptomatic and symptomatic populations. *JAMA Netw Open*. 2020;3(12):e2031517. doi:10.1001/jamanetworkopen.2020.31517

eAppendix. Additional Methods for Study Population, Laboratory Testing, COVID19 Pool Tool, and Virtual Diagnostic Study Analysis

eReference

eFigure 1. Screenshot of COVID19 Pool Tool User Interface

eFigure 2. Example COVID19 Pool Tool Results

This supplemental material has been provided by the authors to give readers additional information about their work.

© 2020 Polage CR et al. *JAMA Network Open.*

eAppendix. Additional Methods for Study Population, Laboratory Testing, COVID19 Pool Tool, and Virtual Diagnostic Study Analysis

Study Population

Retrospective virus copy number (VCN) data from clinical SARS-CoV-2-positive deep nasopharyngeal swab samples from patients tested at the Duke University Health System between March 23rd and July 20th, 2020 was used in the COVID19 Pool Tool and this study. VCNs from pre-procedural screening samples from patients with no clinical suspicion of SARS-CoV-2 coronavirus infection were used to simulate VCNs of asymptomatic patients. These samples were originally collected and tested to document a negative SARS-CoV-2 result within 72 hours before scheduled surgery or procedure unrelated to COVID-19 and not to diagnose COVID-19. Notably, providers were not required to order isolation precautions with these orders unlike other diagnostic orders where clinical suspicion of COVID-19 was generally present to some degree. VCNs from outpatient diagnostic samples from community patients with clinical suspicion for SARS-CoV-2 infection (clinical symptoms of COVID-19 and/or close contact with person(s) with confirmed COVID-19) and indication for diagnostic testing were used to simulate VCNs of symptomatic patients. Other than requirement that VCNs come from pre-procedural screening orders or outpatient diagnostic test orders, there were no eligibility or exclusion criteria and all consecutive positive VCNs from these groups were included. Positive SARS-CoV-2 test results, Ct values, and VCNs were obtained from clinical laboratory databases. Original clinical testing for SARS-CoV-2 detection was performed according to manufacturer's instructions for use using cutoffs for detection and sample positivity defined by the manufacturer and FDA.

© 2020 Polage CR et al. *JAMA Network Open.*

Laboratory Testing

Samples were transported to the laboratory in 3 mL of viral transport media (VTM) or 0.9% sterile saline and tested by one of three FDA Emergency Use Authorized (EUA) SARS-CoV-2 real time reverse transcriptase PCR assays (Abbott® RealTime SARS-CoV-2 RT-PCR, Cepheid® Xpert® Xpress® SARS-CoV-2 RT-PCR, DiaSorin® Simplexa® COVID-19 Direct). Original clinical testing for SARS-CoV-2 detection was performed according to manufacturer's instructions for use using cutoffs for detection and sample positivity defined by the manufacturer and FDA. Invalid samples were retested on a different test platform. SARS-CoV-2 RT-PCR positive patient samples were pooled to create a large volume clinical pool. This clinical pool was serially diluted to create a 10-fold dilution series with multiple aliquots of each dilution. Replicates of each dilution were tested by droplet digital PCR (ddPCR, Bio-Rad AutoDG QX200 System) to create a clinically-derived SARS-CoV-2 ddPCR-quantitated reference standard. Briefly, SARS-CoV-2 RT-PCR positive clinical sample pool aliquots were: 1) extracted with bioMerieux NucliSENS EasyMAG; 2) partitioned into >10,000 single molecule droplets by the AutoDG (BioRad); 3) amplified by RT-qPCR (C100 Touch Thermal Cycler, BioRad) using RNA specific ddPCR SuperMix (1-step RT-ddPCR, BioRad) and CDC 2019-Novel Coronavirus (2019-nCoV) Real-Time RT-PCR Diagnostic Panel primers/probe set (IDT, Inc.); 4) counted by the QX200 Reader (BioRad). Aliquots of this ddPCR-quantitated SARS-CoV-2 reference standard were tested in replicate by and used to create quantitative standard curves for each of the three FDA EUA SARS-CoV-2 RT-PCR clinical assays thereby allowing PCR cycle threshold (Ct) values (results) from all clinical samples (i.e., preoperative screens and outpatient samples) to be converted to a ddPCR-harmonized virus copy number (VCN) per mL of nasopharyngeal swab transport media sample. For samples tested on the Cepheid and DiaSorin platforms, which report results and Ct values for two SARS-CoV-2 targets, both Cts were converted to VCN/mL and the higher of the two VCN values was used for this study; the Abbott RealTime

© 2020 Polage CR et al. *JAMA Network Open.*

Assay detects two targets but only reports a single Ct value (both targets use the same fluorophore), which was used to derive the VCN for samples tested on this platform.

COVID19 Pool Tool

The COVID19 Pool Tool is a web-based interactive tool that allows users to model and understand the expected performance of pooled sample testing for detection of SARS-CoV-2 coronavirus using key model parameters of their choosing. A screenshot of the COVID19 Pool Tool user interface is shown in eFigure 1. Users choose to model detection of asymptomatic or symptomatic SARS-CoV-2 patients as the test population and are given the option to input parameters or choose default settings: 1) the number of samples in the test population; 2) the pool size (i.e., number of samples to be combined in each 'pool'); 3) the expected prevalence or positivity rate in the test population; 4) the 95% confidence interval analytical Limit of Detection (95% LOD) of the SARS-CoV-2 test method being used (available from FDA EUA materials, manufacturer's instructions for use, published material, or local laboratory validation); 5) the absolute LOD (theoretical value used to define lower bound virus copy number (VCN) / mL with approximately 0% chance of detection by test in COVID19 Pool Tool; we set default absolute LOD for the Abbott m2000 RealTime RT-PCR, Cepheid Xpert Xpress, and DiaSorin Simplexa, at to 2, 3.33, and 20 VCN/mL, respectively, based on the sample loading volume of 0.5 mL, 0.3 mL, and 0.05 mL for these tests and stochastic distribution of low copy targets[1]); 6) the number of iterations simulation should run. Based on these parameters, the COVID19 Pool Tool creates random sample sets (i.e., virtual pools) using negative samples and VCN(s) of any positive samples selected randomly from the test population VCN dataset with a probability matching the expected rate of positives in the test population. The pool VCN is calculated for each pool based on the VCN(s) of any positive samples in the pool and number of negative samples in the pool. Virtual/simulated SARS-CoV-2 'testing' of each pool or individual sample uses the following rules: (1) a VCN of 0 results as a 'true negative'; (2) A VCN greater than the

© 2020 Polage CR et al. *JAMA Network Open.*

user-defined 95% LOD results as a 'true positive'; (3) A VCN greater than zero but below the absolute LOD results as a 'false negative'; (4) A VCN between the 95% and absolute LOD has a probability of resulting as 'true positive' that is linearly proportional to its VCN with a 100% chance at the 95% LOD and a 0% chance at the absolute LOD. Samples in 'true positive' pools are virtually retested as individual samples (single samples) using the same rules (i.e., the COVID19 Pool Tool pooled testing strategy uses a Dorfman-style approach where positive pools are deconvoluted and have individual samples retested as single samples). The simulation counts the total number of tests performed (number of pools tested + number of individual samples retested from 'true positive' pools to identify individual positive samples) and the number and percent of 'true positives' over 'true positive' + 'false negative' results (sensitivity). For comparison, the COVID19 Pool Tool performs virtual/simulation SARS-CoV-2 'testing' on all individual samples included in pools by a single sample testing strategy using the same resulting rules. An example of COVID19 Pool Tool results is shown in eFigure 2. All COVID19 Pool Tool results are reported as calculated value ± standard deviation.

Virtual Diagnostic Study Analysis

Each run of the COVID19 Pool Tool is a virtual diagnostic study comparing the performance of two candidate tests (user-defined pooled testing strategy and user-defined single sample test) to detect known positive clinical samples individually or within randomly created virtual pools (reference standard). VCNs from clinical pre-procedural screening samples are used to simulate asymptomatic SARS-CoV-2-positive patients; VCNs from outpatient diagnostic samples are used to simulate symptomatic SARS-CoV-2-positive patients. For this study, we ran the COVID19 Pool Tool at six escalating positivity rates with asymptomatic VCNs (0.1%, 1%, 5%, 10%, 15%, 20%) and again with the same six escalating positivity rates with symptomatic VCNs (total of 12 runs). For each run, we used n=1000 individual samples (i.e., virtual diagnostic comparison study of n=1000 samples for each positivity rate and VCN patient population), pool

© 2020 Polage CR et al. *JAMA Network Open.*

size = 100 samples, analytical sensitivity test characteristics for the Abbott RealTime SARS-CoV-2 Assay (95% LoD – 100 copies/mL; absolute LoD – 2 copies/mL), and 10,000 iterations. The output of each run provides results for single sample testing and all pool sizes from 2-100 samples combined in the pool. The results from these runs allowed us to compare and plot the results in Figure 2a-2f: the number of false negative cases per 1,000 patients screened (a measure of sensitivity); the number of tests performed per 1,000 patients screened (a measure of testing scheme efficiency); the number of SARS-CoV-2 cases identified per 1,000 tests performed (a measure of overall effectiveness combining test sensitivity and efficiency of testing scheme). For each graph within Figure 2a-2f, results of single sample testing are displayed on the Y-axis where Pool size equals 1 and pooled sample testing results are displayed across the X-axis for Pool sizes 2-64+.

© 2020 Polage CR et al. *JAMA Network Open.*

eReference

1. Stephen A. Bustin SA, Benes V, Garson JA, et al. The MIQE Guidelines: Minimum Information for Publication of Quantitative Real-Time PCR Experiments. Clin Chem 2009 55(4):611–622.

© 2020 Polage CR et al. *JAMA Network Open.*

eFigure 1. Screenshot of COVID19 Pool Tool User Interface

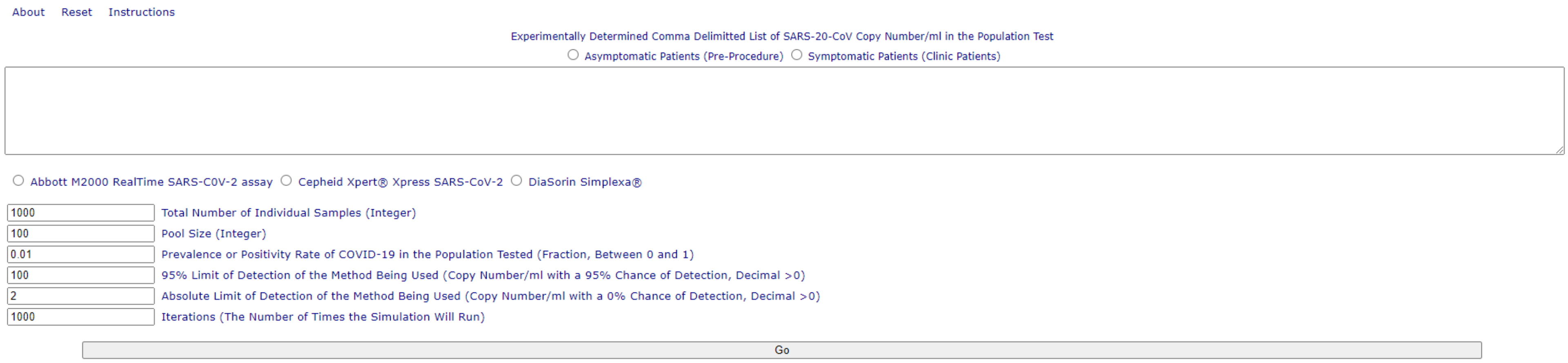

© 2020 Polage CR et al. *JAMA Network Open.*

eFigure 2. Example COVID19 Pool Tool Results

Experimentally Determined Comma Delimitted List of SARS-20-CoV Copy Number/ml in the Population Test

◉ Asymptomatic Patients (Pre-Procedure) ○ Symptomatic Patients (Clinic Patients)

75800,79100000,6.12,1770000,123,370,17800000,112,283,140000000,821,190,4560000,515,673,395,768,99600000,582,1710,1950,58.9,6400000,482,8.89,1160000,248,22100,32000,7850000,496,451000000,311,291,65400000,6.52,366,12600,16600,65.5,104,303,58.9,3660,183,8
140,183,67.3,283,55.1,37700,24800000,160,55.1,291,71.9,4180,21100000,11800,19700000,5120000,5290,11600000,110,107,140,832000,2470,415,67300000,56300000,60200000,28000,206000

◉ Abbott M2000 RealTime SARS-COV-2 assay ○ Cepheid Xpert® Xpress SARS-CoV-2 ○ DiaSorin Simplexa®

1000 Total Number of Individual Samples (Integer)
100 Pool Size (Integer)
0.01 Prevalence or Positivity Rate of COVID-19 in the Population Tested (Fraction, Between 0 and 1)
100 95% Limit of Detection of the Method Being Used (Copy Number/ml with a 95% Chance of Detection, Decimal >0)
2 Absolute Limit of Detection of the Method Being Used (Copy Number/ml with a 0% Chance of Detection, Decimal >0)
1000 Iterations (The Number of Times the Simulation Will Run)

Go

Analysis Started: **7/29/2020, 3:46:44 PM**
Total Number of Individual Samples: **1000**
Pool Size: **100**
Prevalence or Positivity Rate of COVID-19 in the Population Tested (Fraction, Between 0 and 1): **0.01**
95% Limit of Detection of the Method Being Used (Copy Number/ml with a 95% Chance of Detection, Decimal >0): **100**
Absolute Limit of Detection of the Method Being Used (Copy Number/ml with a 0% Chance of Detection, Decimal >0): **2**
Iterations (The Number of Times the Simulation Will Run): **1000**

Sample Count	**Pool Size**	Total Pools	Average Positive Samples	Avearge Negative Pools (no Virus Present)	Average Positive Pools (Virus Present At Any Level)	Average Samples Below 95% LOD	Average Pools Below 95% LOD	Average False Negative Pools	Percent False Negative Pools	Average False Negative Results (Pool)	**Average Sensitivty (Pool)**	Average False Negative Results (No Pool)	**Average Sensitivity (No Pool)**	Average Tests Performed	**Average Percent Performed**
1000	**2**	500	10.10±3.20	489.95±3.19	10.05±3.19	1.36±1.17	2.67±1.69	1.44±1.23	14.29%±12.28	1.57±1.28	**84.38% ±12.66**	0.76±0.89	**92.48% ±9.04**	517.2±5.9	**51.72%±0.59**
1000	**3**	334	9.89±3.20	324.20±3.16	9.80±3.16	1.36±1.23	3.28±1.85	1.88±1.43	19.02%±13.52	1.98±1.47	**80.14% ±13.68**	0.77±0.90	**92.31% ±9.07**	357.7±8.5	**35.77%±0.85**
1000	**4**	250	9.89±3.29	240.26±3.21	9.74±3.21	1.35±1.18	3.90±2.02	2.29±1.55	23.20%±14.37	2.36±1.56	**76.34% ±14.29**	0.74±0.85	**92.54% ±8.64**	279.8±11.0	**27.98%±1.10**
1000	**5**	200	10.10±3.24	190.10±3.12	9.90±3.12	1.37±1.16	4.39±2.00	2.83±1.58	28.62%±14.32	2.89±1.60	**71.22% ±14.31**	0.74±0.87	**92.73% ±8.73**	235.3±13.2	**23.54%±1.32**
1000	**6**	167	10.21±3.11	157.06±3.01	9.94±3.01	1.34±1.14	4.57±2.08	2.96±1.71	29.71%±15.33	3.02±1.74	**70.36% ±15.34**	0.78±0.86	**92.38% ±8.71**	208.8±15.3	**20.88%±1.53**
1000	**7**	143	10.07±3.20	133.24±3.04	9.76±3.04	1.39±1.14	4.62±2.14	3.19±1.77	32.74%±16.19	3.26±1.80	**67.39% ±16.12**	0.76±0.88	**92.31% ±9.05**	189.0±17.6	**18.90%±1.76**
1000	**8**	125	10.04±3.20	115.31±3.07	9.69±3.07	1.31±1.13	4.76±2.18	3.40±1.85	34.94%±16.47	3.48±1.88	**65.32% ±16.38**	0.72±0.86	**92.73% ±9.11**	175.4±20.2	**17.54%±2.02**
1000	**9**	112	9.94±3.10	102.42±2.91	9.57±2.91	1.38±1.20	4.85±2.13	3.57±1.84	37.10%±15.99	3.67±1.91	**63.22% ±16.03**	0.77±0.89	**92.37% ±8.75**	166.0±20.9	**16.60%±2.09**
1000	**10**	100	9.93±3.18	90.51±2.97	9.49±2.97	1.32±1.15	4.74±2.11	3.55±1.83	37.45%±16.10	3.65±1.91	**62.96% ±16.27**	0.73±0.86	**92.71% ±8.71**	159.5±23.9	**15.95%±2.39**

© 2020 Polage CR et al. *JAMA Network Open.*
